# 1-(4-Methyl­benzo­yl)-3-{2-[3-(4-methyl­benzo­yl)thio­ureido]phen­yl}thio­urea

**DOI:** 10.1107/S1600536811035586

**Published:** 2011-09-14

**Authors:** Uwaisulqarni M. Osman, Bohari M. Yamin

**Affiliations:** a School of Chemical Sciences and Food Technology, Universiti Kebangsaan Malaysia, UKM 43500 Bangi Selangor, Malaysia

## Abstract

In the title compound, C_24_H_22_N_4_O_2_S_2_, the dihedral angles formed by the thio­ureido groups with the attached benzene ring are 43.81 (13) and 75.25 (13)°. The dihedral angle between the thio­ureido groups is 85.48 (10)°. The mol­ecule is stabilized by intra­molecular N—H⋯S, N—H⋯O and C—H⋯S hydrogen bonds. In the crystal, molecules are linked by intermolecular N—H⋯S hydrogen bonds together with C—H⋯π inter­actions.

## Related literature

For the structure of related bis-carbomothioyl thio­ureas, see: Yamin & Osman (2011 ▶); Thiam *et al.* (2008 ▶). 
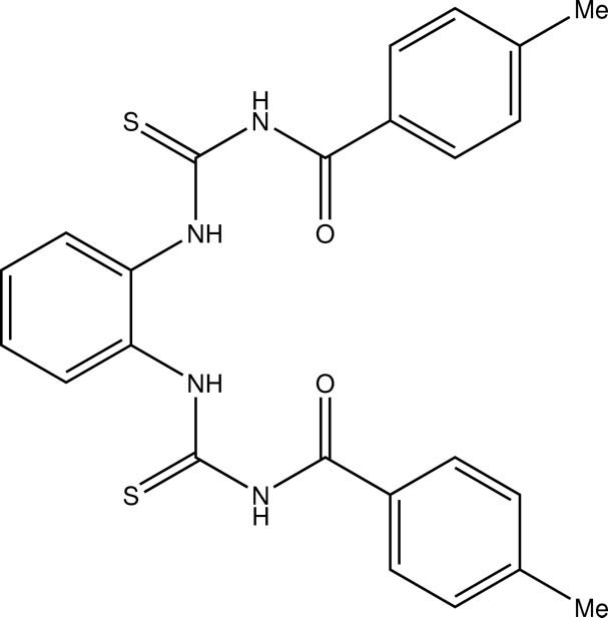


## Experimental

### 

#### Crystal data

C_24_H_22_N_4_O_2_S_2_
*M* *_r_* = 462.58
Triclinic, 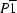

*a* = 7.1565 (18) Å
*b* = 11.394 (3) Å
*c* = 14.332 (4) Å
α = 96.414 (5)°
β = 99.066 (6)°
γ = 94.085 (6)°
*V* = 1142.1 (5) Å^3^
*Z* = 2
Mo *K*α radiation
μ = 0.26 mm^−1^
*T* = 298 K
0.50 × 0.12 × 0.06 mm

#### Data collection

Bruker SMART APEX CCD area-detector diffractometer
Absorption correction: multi-scan (*SADABS*; Bruker, 2000 ▶) *T*_min_ = 0.880, *T*_max_ = 0.984
13125 measured reflections
4472 independent reflections
2810 reflections with *I* > 2σ(*I*)
*R*_int_ = 0.053

#### Refinement

*R*[*F* ^2^ > 2σ(*F* ^2^)] = 0.064
*wR*(*F* ^2^) = 0.143
*S* = 1.02
4472 reflections
294 parameters
1 restraint
H atoms treated by a mixture of independent and constrained refinement
Δρ_max_ = 0.32 e Å^−3^
Δρ_min_ = −0.19 e Å^−3^

### 

Data collection: *SMART* (Bruker, 2000 ▶); cell refinement: *SAINT* (Bruker, 2000 ▶); data reduction: *SAINT*; program(s) used to solve structure: *SHELXTL* (Sheldrick, 2008 ▶); program(s) used to refine structure: *SHELXTL*; molecular graphics: *SHELXTL*; software used to prepare material for publication: *SHELXTL*, *PARST* (Nardelli, 1995 ▶) and *PLATON* (Spek, 2009 ▶).

## Supplementary Material

Crystal structure: contains datablock(s) global, I. DOI: 10.1107/S1600536811035586/rz2633sup1.cif

Structure factors: contains datablock(s) I. DOI: 10.1107/S1600536811035586/rz2633Isup2.hkl

Supplementary material file. DOI: 10.1107/S1600536811035586/rz2633Isup3.cml

Additional supplementary materials:  crystallographic information; 3D view; checkCIF report

## Figures and Tables

**Table 1:** Hydrogen-bond geometry (Å, °) *Cg*1 is the centroid of the C9–C14 ring.

*D*—H⋯*A*	*D*—H	H⋯*A*	*D*⋯*A*	*D*—H⋯*A*
N1—H1⋯S2	0.86	2.83	3.476 (3)	134
N1—H1⋯O1	0.86	1.95	2.651 (3)	138
N3—H3⋯O2	0.86	1.97	2.640 (3)	134
C2—H2*A*⋯S1	0.93	2.79	3.223 (3)	110
N4—H4⋯S2^i^	0.86	2.71	3.533 (3)	161
C15—H15*B*⋯*Cg*1^ii^	0.96	2.76	3.509 (4)	136

Symmetry codes: (i) 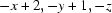; (ii) 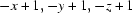.

**Fig. 1.**
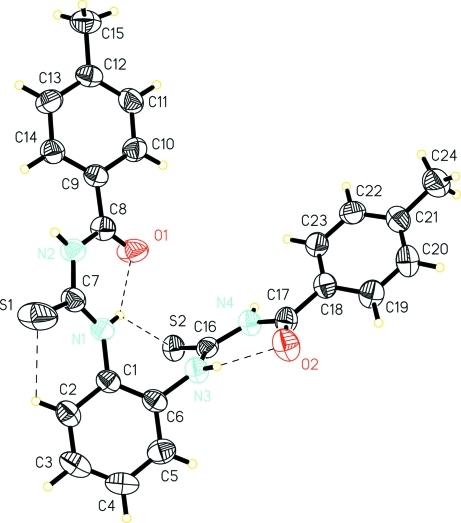
The molecular structure of the title compound, with displacement ellipsoids drawn at the 50% probability level. Intermolecular hydrogen bonds are shown as dashed lines.

**Fig. 2.**
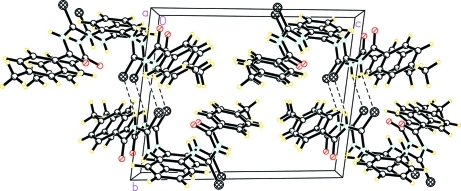
A packing diagram of the title compound viewed along the a axis. Hydrogen bonds are shown as dashed lines.

## supplementary crystallographic information

### Comment

The title compound, 1,2-bis(*N*'-4-methylbenzoylthioureido)benzene (systematic name: 1-(4-methylbenzoyl)-3-{2-[3-(4-methylbenzoyl)thioureido]phenyl}thiourea), is similar to 1,2-bis(*N*'-benzoylthioureido)benzene (Thiam *et al.*, 2008) except for the presence of methyl groups at *para* position of the benzoyl group (Fig. 1). Both thioureido groups S1/N1/N2/C7/C1 and S2/N3/N4/C16/C6 are planar with maximum deviation from the least square planes of 0.033 (2)Å for the N1 atom. The thioureido groups form dihedral angles of 43.81 (13) and 75.25 (13) Å, respectively, with the central benzene ring. The dihedral between the two thioureido groups is 85.58 (10)°. There are four intramolecular hydrogen bonds forming three six-membered ring [O1···H1—N1—C7—N2—C8], [O2···H3A—N3—C16—N4—C17] and [H2A···S1—C7—N1—C1—C2], and one seven-membered ring [H1···S2—C16—N3—C6—C1—N1] as compared to two intramolecular hydrogen bonds observed in 1,2-bis(*N*'-benzoylthioureido) benzene. The introduction of chloro atom to the bridging benzene ring in 1,2-bis(*N*'-benzoylthioureido)-4-chlorobenzene (Yamin & Osman, 2011) allowed four intramolecular hydrogen bonds. In the crystal structure, the molecules are linked by N1—H1A···S1 intermolecular hydrogen bonds (symmetry codes as in Table 1) to form centrosymmetric dimers (Fig. 2). In addition, a C—H···π interaction with distance of 2.760Å and an angle of 136° is also present.

### Experimental

To a stirred acetone solution (75 ml) of *para*-benzoyl chloride (0.04 mol) and ammonium thiocyanate (0.04 mol) 1,2-phenylenediamine (0.02 mol) in 40 ml of acetone was added dropwise. The solution mixture was refluxed for 1 h. The resulting solution was poured into a beaker containing some ice cubes. The white precipitate formed was filtered off, washed with distilled water and cold ethanol and then dried under vacuum. Good quality crystals were obtained by recrystallization from ethanol.

### Refinement

The hydrogen atom attached to the N2 atom was refined freely, with the N—H distance restrained to be 0.86 Å and *U*_iso_(H) = 1.2 *U*_eq_(N). All other H atoms were positioned geometrically and constrained to ride on their parent atoms, with C—H(aromatic) = 0.93 Å, *C*—*H*(alkyl) = 0.96 Å, N—H = 0.86 Å, and with *U*_iso_(H) = *x U*_eq_(parent atom) where *x* = 1.2 for both CH(aromatic) and NH groups, and *x* = 1.5 for CH(methyl) groups. A rotating group model was applied to the methyl groups.

### Crystal data

**Table:**

C_24_H_22_N_4_O_2_S_2_	*Z* = 2
*M**_r_* = 462.58	*F*(000) = 484
Triclinic, *P*1	*D*_x_ = 1.345 Mg m^−^^3^
Hall symbol: -P 1	Melting point = 475.4–476.6 K
*a* = 7.1565 (18) Å	Mo *K*α radiation, λ = 0.71073 Å
*b* = 11.394 (3) Å	Cell parameters from 1550 reflections
*c* = 14.332 (4) Å	θ = 1.8–26.0°
α = 96.414 (5)°	µ = 0.26 mm^−^^1^
β = 99.066 (6)°	*T* = 298 K
γ = 94.085 (6)°	Slab, colourless
*V* = 1142.1 (5) Å^3^	0.50 × 0.12 × 0.06 mm

### Data collection

**Table:**

Bruker SMART APEX CCD area-detector diffractometer	4472 independent reflections
Radiation source: fine-focus sealed tube	2810 reflections with *I* > 2σ(*I*)
graphite	*R*_int_ = 0.053
Detector resolution: 83.66 pixels mm^-1^	θ_max_ = 26.0°, θ_min_ = 1.8°
ω scans	*h* = −8→8
Absorption correction: multi-scan (*SADABS*; Bruker, 2000)	*k* = −14→14
*T*_min_ = 0.880, *T*_max_ = 0.984	*l* = −17→17
13125 measured reflections	

### Refinement

**Table:**

Refinement on *F*^2^	Primary atom site location: structure-invariant direct methods
Least-squares matrix: full	Secondary atom site location: difference Fourier map
*R*[*F*^2^ > 2σ(*F*^2^)] = 0.064	Hydrogen site location: inferred from neighbouring sites
*wR*(*F*^2^) = 0.143	H atoms treated by a mixture of independent and constrained refinement
*S* = 1.02	*w* = 1/[σ^2^(*F*_o_^2^) + (0.0601*P*)^2^ + 0.1876*P*] where *P* = (*F*_o_^2^ + 2*F*_c_^2^)/3
4472 reflections	(Δ/σ)_max_ < 0.001
294 parameters	Δρ_max_ = 0.32 e Å^−^^3^
1 restraint	Δρ_min_ = −0.19 e Å^−^^3^

### Special details

**Table:**

Geometry. All e.s.d.'s (except the e.s.d. in the dihedral angle between two l.s. planes) are estimated using the full covariance matrix. The cell e.s.d.'s are taken into account individually in the estimation of e.s.d.'s in distances, angles and torsion angles; correlations between e.s.d.'s in cell parameters are only used when they are defined by crystal symmetry. An approximate (isotropic) treatment of cell e.s.d.'s is used for estimating e.s.d.'s involving l.s. planes.
Refinement. Refinement of *F*^2^ against ALL reflections. The weighted *R*-factor *wR* and goodness of fit *S* are based on *F*^2^, conventional *R*-factors *R* are based on *F*, with *F* set to zero for negative *F*^2^. The threshold expression of *F*^2^ > σ(*F*^2^) is used only for calculating *R*-factors(gt) *etc*. and is not relevant to the choice of reflections for refinement. *R*-factors based on *F*^2^ are statistically about twice as large as those based on *F*, and *R*- factors based on ALL data will be even larger.

### Fractional atomic coordinates and isotropic or equivalent isotropic displacement parameters (Å^2^)

**Table:**

	*x*	*y*	*z*	*U*_iso_*/*U*_eq_	
S1	1.23642 (15)	1.02145 (9)	0.41453 (8)	0.0810 (4)	
S2	1.21023 (12)	0.60423 (7)	0.10031 (6)	0.0488 (3)	
O1	0.9229 (3)	0.6795 (2)	0.25909 (17)	0.0661 (7)	
O2	0.9013 (3)	0.87729 (19)	−0.05615 (16)	0.0638 (7)	
N1	1.2155 (3)	0.8412 (2)	0.27623 (17)	0.0438 (6)	
H1	1.1524	0.7764	0.2483	0.053*	
N2	1.0044 (4)	0.8279 (2)	0.38102 (18)	0.0457 (6)	
H2	0.968 (4)	0.868 (2)	0.4283 (14)	0.055*	
N3	1.1759 (3)	0.8295 (2)	0.07633 (17)	0.0467 (6)	
H3	1.1088	0.8820	0.0526	0.056*	
N4	0.9377 (3)	0.6947 (2)	−0.00916 (17)	0.0447 (6)	
H4	0.8882	0.6224	−0.0191	0.054*	
C1	1.3716 (4)	0.8820 (2)	0.2346 (2)	0.0403 (7)	
C2	1.5444 (4)	0.9285 (3)	0.2888 (2)	0.0489 (8)	
H2A	1.5595	0.9358	0.3549	0.059*	
C3	1.6929 (5)	0.9638 (3)	0.2455 (3)	0.0566 (9)	
H3A	1.8068	0.9970	0.2826	0.068*	
C4	1.6755 (5)	0.9508 (3)	0.1478 (3)	0.0598 (9)	
H4A	1.7777	0.9736	0.1190	0.072*	
C5	1.5054 (5)	0.9036 (3)	0.0931 (2)	0.0534 (8)	
H5	1.4926	0.8943	0.0270	0.064*	
C6	1.3535 (4)	0.8699 (2)	0.1363 (2)	0.0418 (7)	
C7	1.1550 (4)	0.8911 (3)	0.3530 (2)	0.0456 (8)	
C8	0.8930 (4)	0.7289 (3)	0.3340 (2)	0.0454 (8)	
C9	0.7318 (4)	0.6900 (2)	0.3800 (2)	0.0416 (7)	
C10	0.5718 (4)	0.6305 (3)	0.3223 (2)	0.0509 (8)	
H10	0.5712	0.6124	0.2573	0.061*	
C11	0.4137 (5)	0.5981 (3)	0.3605 (2)	0.0557 (9)	
H11	0.3079	0.5575	0.3209	0.067*	
C12	0.4092 (4)	0.6245 (3)	0.4563 (2)	0.0475 (8)	
C13	0.5708 (5)	0.6804 (3)	0.5143 (2)	0.0541 (9)	
H13	0.5722	0.6965	0.5795	0.065*	
C14	0.7305 (4)	0.7125 (3)	0.4766 (2)	0.0492 (8)	
H14	0.8382	0.7498	0.5167	0.059*	
C15	0.2339 (5)	0.5942 (3)	0.4979 (3)	0.0646 (10)	
H15A	0.1803	0.6658	0.5178	0.097*	
H15B	0.2671	0.5523	0.5517	0.097*	
H15C	0.1425	0.5452	0.4506	0.097*	
C16	1.1071 (4)	0.7164 (3)	0.05479 (19)	0.0409 (7)	
C17	0.8385 (5)	0.7739 (3)	−0.0587 (2)	0.0466 (8)	
C18	0.6482 (4)	0.7287 (3)	−0.1130 (2)	0.0442 (7)	
C19	0.5775 (5)	0.7814 (3)	−0.1920 (2)	0.0528 (9)	
H19	0.6531	0.8397	−0.2126	0.063*	
C20	0.3966 (5)	0.7482 (3)	−0.2401 (2)	0.0578 (9)	
H20	0.3527	0.7835	−0.2939	0.069*	
C21	0.2779 (5)	0.6637 (3)	−0.2106 (2)	0.0513 (8)	
C22	0.3506 (5)	0.6101 (3)	−0.1326 (2)	0.0547 (9)	
H22	0.2750	0.5515	−0.1123	0.066*	
C23	0.5325 (5)	0.6415 (3)	−0.0843 (2)	0.0503 (8)	
H23	0.5782	0.6039	−0.0320	0.060*	
C24	0.0761 (5)	0.6314 (4)	−0.2600 (3)	0.0761 (11)	
H24A	0.0074	0.5885	−0.2203	0.114*	
H24B	0.0755	0.5828	−0.3193	0.114*	
H24C	0.0167	0.7024	−0.2721	0.114*	

### Atomic displacement parameters (Å^2^)

**Table:**

	*U*^11^	*U*^22^	*U*^33^	*U*^12^	*U*^13^	*U*^23^
S1	0.0742 (7)	0.0602 (6)	0.1025 (8)	−0.0252 (5)	0.0373 (6)	−0.0290 (6)
S2	0.0552 (5)	0.0405 (5)	0.0473 (5)	0.0033 (4)	−0.0002 (4)	0.0039 (4)
O1	0.0648 (16)	0.0619 (15)	0.0678 (16)	−0.0208 (12)	0.0280 (13)	−0.0159 (13)
O2	0.0678 (16)	0.0420 (13)	0.0732 (16)	−0.0071 (12)	−0.0106 (13)	0.0107 (11)
N1	0.0438 (15)	0.0411 (14)	0.0438 (15)	−0.0100 (12)	0.0060 (12)	0.0034 (12)
N2	0.0462 (16)	0.0428 (15)	0.0469 (16)	−0.0064 (12)	0.0118 (13)	0.0008 (12)
N3	0.0494 (16)	0.0390 (15)	0.0488 (16)	−0.0002 (12)	0.0002 (13)	0.0070 (12)
N4	0.0502 (16)	0.0328 (13)	0.0467 (15)	−0.0025 (12)	−0.0009 (13)	0.0022 (11)
C1	0.0391 (18)	0.0312 (15)	0.0496 (19)	−0.0006 (13)	0.0049 (15)	0.0065 (13)
C2	0.0435 (19)	0.0444 (18)	0.055 (2)	−0.0012 (15)	−0.0011 (16)	0.0055 (15)
C3	0.0372 (19)	0.0426 (19)	0.088 (3)	0.0026 (15)	0.0047 (19)	0.0097 (18)
C4	0.046 (2)	0.054 (2)	0.085 (3)	0.0001 (17)	0.026 (2)	0.0114 (19)
C5	0.058 (2)	0.0463 (19)	0.059 (2)	0.0030 (17)	0.0173 (18)	0.0077 (16)
C6	0.0395 (18)	0.0321 (16)	0.052 (2)	−0.0014 (13)	0.0055 (15)	0.0043 (14)
C7	0.0423 (18)	0.0441 (18)	0.049 (2)	−0.0020 (15)	0.0043 (15)	0.0058 (15)
C8	0.0479 (19)	0.0369 (17)	0.050 (2)	0.0011 (15)	0.0086 (16)	0.0014 (15)
C9	0.0405 (18)	0.0346 (16)	0.0495 (19)	0.0004 (14)	0.0083 (15)	0.0059 (14)
C10	0.053 (2)	0.0518 (19)	0.0471 (19)	−0.0055 (16)	0.0095 (17)	0.0091 (15)
C11	0.046 (2)	0.059 (2)	0.058 (2)	−0.0091 (17)	0.0009 (17)	0.0098 (17)
C12	0.0408 (19)	0.0436 (18)	0.062 (2)	0.0070 (15)	0.0136 (16)	0.0132 (16)
C13	0.057 (2)	0.056 (2)	0.051 (2)	−0.0006 (17)	0.0200 (18)	0.0030 (16)
C14	0.0460 (19)	0.0492 (19)	0.049 (2)	−0.0034 (15)	0.0070 (16)	−0.0034 (15)
C15	0.050 (2)	0.072 (2)	0.078 (3)	0.0047 (18)	0.0217 (19)	0.022 (2)
C16	0.0430 (18)	0.0446 (18)	0.0347 (17)	−0.0015 (14)	0.0105 (14)	0.0012 (14)
C17	0.054 (2)	0.0427 (19)	0.0406 (18)	0.0033 (16)	0.0030 (15)	0.0030 (15)
C18	0.0486 (19)	0.0390 (17)	0.0423 (18)	0.0040 (15)	0.0038 (15)	−0.0015 (14)
C19	0.065 (2)	0.0398 (18)	0.050 (2)	−0.0036 (16)	0.0046 (18)	0.0063 (15)
C20	0.068 (2)	0.049 (2)	0.051 (2)	0.0066 (18)	−0.0066 (18)	0.0041 (16)
C21	0.050 (2)	0.054 (2)	0.046 (2)	0.0082 (17)	0.0033 (16)	−0.0092 (16)
C22	0.052 (2)	0.060 (2)	0.052 (2)	−0.0020 (17)	0.0153 (17)	0.0035 (17)
C23	0.050 (2)	0.056 (2)	0.0448 (19)	0.0074 (17)	0.0068 (16)	0.0087 (15)
C24	0.059 (2)	0.101 (3)	0.063 (2)	0.002 (2)	0.0022 (19)	−0.001 (2)

### Geometric parameters (Å, °)

**Table:**

S1—C7	1.656 (3)	C9—C10	1.386 (4)
S2—C16	1.665 (3)	C10—C11	1.378 (4)
O1—C8	1.213 (3)	C10—H10	0.9300
O2—C17	1.225 (3)	C11—C12	1.377 (4)
N1—C7	1.329 (4)	C11—H11	0.9300
N1—C1	1.421 (4)	C12—C13	1.382 (4)
N1—H1	0.8600	C12—C15	1.506 (4)
N2—C8	1.377 (4)	C13—C14	1.383 (4)
N2—C7	1.390 (4)	C13—H13	0.9300
N2—H2	0.859 (10)	C14—H14	0.9300
N3—C16	1.331 (3)	C15—H15A	0.9600
N3—C6	1.432 (4)	C15—H15B	0.9600
N3—H3	0.8600	C15—H15C	0.9600
N4—C17	1.378 (4)	C17—C18	1.484 (4)
N4—C16	1.389 (4)	C18—C19	1.382 (4)
N4—H4	0.8600	C18—C23	1.387 (4)
C1—C6	1.385 (4)	C19—C20	1.373 (4)
C1—C2	1.387 (4)	C19—H19	0.9300
C2—C3	1.371 (4)	C20—C21	1.384 (5)
C2—H2A	0.9300	C20—H20	0.9300
C3—C4	1.376 (5)	C21—C22	1.380 (4)
C3—H3A	0.9300	C21—C24	1.506 (4)
C4—C5	1.378 (4)	C22—C23	1.378 (4)
C4—H4A	0.9300	C22—H22	0.9300
C5—C6	1.385 (4)	C23—H23	0.9300
C5—H5	0.9300	C24—H24A	0.9600
C8—C9	1.481 (4)	C24—H24B	0.9600
C9—C14	1.381 (4)	C24—H24C	0.9600
			
C7—N1—C1	127.9 (2)	C11—C12—C13	118.3 (3)
C7—N1—H1	116.1	C11—C12—C15	121.6 (3)
C1—N1—H1	116.1	C13—C12—C15	120.1 (3)
C8—N2—C7	129.1 (3)	C12—C13—C14	120.7 (3)
C8—N2—H2	120 (2)	C12—C13—H13	119.6
C7—N2—H2	110 (2)	C14—C13—H13	119.6
C16—N3—C6	124.7 (3)	C9—C14—C13	120.8 (3)
C16—N3—H3	117.7	C9—C14—H14	119.6
C6—N3—H3	117.7	C13—C14—H14	119.6
C17—N4—C16	128.4 (3)	C12—C15—H15A	109.5
C17—N4—H4	115.8	C12—C15—H15B	109.5
C16—N4—H4	115.8	H15A—C15—H15B	109.5
C6—C1—C2	118.8 (3)	C12—C15—H15C	109.5
C6—C1—N1	118.7 (3)	H15A—C15—H15C	109.5
C2—C1—N1	122.4 (3)	H15B—C15—H15C	109.5
C3—C2—C1	120.4 (3)	N3—C16—N4	115.9 (3)
C3—C2—H2A	119.8	N3—C16—S2	124.1 (2)
C1—C2—H2A	119.8	N4—C16—S2	120.0 (2)
C2—C3—C4	120.8 (3)	O2—C17—N4	121.9 (3)
C2—C3—H3A	119.6	O2—C17—C18	121.5 (3)
C4—C3—H3A	119.6	N4—C17—C18	116.6 (3)
C3—C4—C5	119.4 (3)	C19—C18—C23	118.4 (3)
C3—C4—H4A	120.3	C19—C18—C17	118.6 (3)
C5—C4—H4A	120.3	C23—C18—C17	122.8 (3)
C4—C5—C6	120.1 (3)	C20—C19—C18	120.5 (3)
C4—C5—H5	119.9	C20—C19—H19	119.7
C6—C5—H5	119.9	C18—C19—H19	119.7
C5—C6—C1	120.4 (3)	C19—C20—C21	121.6 (3)
C5—C6—N3	117.9 (3)	C19—C20—H20	119.2
C1—C6—N3	121.5 (3)	C21—C20—H20	119.2
N1—C7—N2	115.5 (3)	C22—C21—C20	117.5 (3)
N1—C7—S1	126.3 (2)	C22—C21—C24	120.6 (3)
N2—C7—S1	118.1 (2)	C20—C21—C24	121.9 (3)
O1—C8—N2	121.9 (3)	C23—C22—C21	121.5 (3)
O1—C8—C9	122.9 (3)	C23—C22—H22	119.3
N2—C8—C9	115.2 (3)	C21—C22—H22	119.3
C14—C9—C10	118.4 (3)	C22—C23—C18	120.4 (3)
C14—C9—C8	123.6 (3)	C22—C23—H23	119.8
C10—C9—C8	117.9 (3)	C18—C23—H23	119.8
C11—C10—C9	120.4 (3)	C21—C24—H24A	109.5
C11—C10—H10	119.8	C21—C24—H24B	109.5
C9—C10—H10	119.8	H24A—C24—H24B	109.5
C12—C11—C10	121.3 (3)	C21—C24—H24C	109.5
C12—C11—H11	119.3	H24A—C24—H24C	109.5
C10—C11—H11	119.3	H24B—C24—H24C	109.5
			
C7—N1—C1—C6	−141.4 (3)	C10—C11—C12—C13	−2.6 (5)
C7—N1—C1—C2	41.6 (4)	C10—C11—C12—C15	177.2 (3)
C6—C1—C2—C3	1.3 (4)	C11—C12—C13—C14	2.2 (5)
N1—C1—C2—C3	178.2 (3)	C15—C12—C13—C14	−177.6 (3)
C1—C2—C3—C4	−1.9 (5)	C10—C9—C14—C13	−2.3 (4)
C2—C3—C4—C5	1.1 (5)	C8—C9—C14—C13	175.9 (3)
C3—C4—C5—C6	0.1 (5)	C12—C13—C14—C9	0.3 (5)
C4—C5—C6—C1	−0.7 (5)	C6—N3—C16—N4	−176.2 (3)
C4—C5—C6—N3	175.5 (3)	C6—N3—C16—S2	4.4 (4)
C2—C1—C6—C5	0.0 (4)	C17—N4—C16—N3	5.3 (4)
N1—C1—C6—C5	−177.0 (3)	C17—N4—C16—S2	−175.3 (2)
C2—C1—C6—N3	−176.1 (3)	C16—N4—C17—O2	5.6 (5)
N1—C1—C6—N3	6.9 (4)	C16—N4—C17—C18	−172.4 (3)
C16—N3—C6—C5	103.2 (3)	O2—C17—C18—C19	28.5 (5)
C16—N3—C6—C1	−80.6 (4)	N4—C17—C18—C19	−153.4 (3)
C1—N1—C7—N2	−176.4 (3)	O2—C17—C18—C23	−146.9 (3)
C1—N1—C7—S1	5.9 (5)	N4—C17—C18—C23	31.1 (4)
C8—N2—C7—N1	−9.3 (5)	C23—C18—C19—C20	0.4 (5)
C8—N2—C7—S1	168.5 (3)	C17—C18—C19—C20	−175.2 (3)
C7—N2—C8—O1	3.7 (5)	C18—C19—C20—C21	1.4 (5)
C7—N2—C8—C9	−174.4 (3)	C19—C20—C21—C22	−2.4 (5)
O1—C8—C9—C14	155.4 (3)	C19—C20—C21—C24	176.8 (3)
N2—C8—C9—C14	−26.5 (4)	C20—C21—C22—C23	1.7 (5)
O1—C8—C9—C10	−26.4 (4)	C24—C21—C22—C23	−177.6 (3)
N2—C8—C9—C10	151.7 (3)	C21—C22—C23—C18	0.1 (5)
C14—C9—C10—C11	1.9 (5)	C19—C18—C23—C22	−1.1 (5)
C8—C9—C10—C11	−176.4 (3)	C17—C18—C23—C22	174.3 (3)
C9—C10—C11—C12	0.6 (5)		

### Hydrogen-bond geometry (Å, °)

**Table:**

*Cg*1 is the centroid of the C9–C14 ring.

**Table:**

*D*—H···*A*	*D*—H	H···*A*	*D*···*A*	*D*—H···*A*
N1—H1···S2	0.86	2.83	3.476 (3)	134
N1—H1···O1i	0.86	1.95	2.651 (3)	138
N3—H3···O2	0.86	1.97	2.640 (3)	134
C2—H2A···S1	0.93	2.79	3.223 (3)	110
N4—H4···S2^i^	0.86	2.71	3.533 (3)	161
C15—H15B···Cg1^ii^	0.96	2.76	3.509 (4)	136

Symmetry codes:  i; (i) −*x*+2, −*y*+1, −*z*; (ii) −*x*+1, −*y*+1, −*z*+1.
